# Applying a synergistic mindsets intervention to an esports context

**DOI:** 10.1098/rsos.240691

**Published:** 2024-06-26

**Authors:** Maciej Behnke, Daniel Lakens, Kate Petrova, Patrycja Chwiłkowska, Szymon Jęśko Białek, Maciej Kłoskowski, Wadim Krzyżaniak, Patryk Maciejewski, Lukasz D. Kaczmarek, Kacper Szymański, Jeremy P. Jamieson, James J. Gross

**Affiliations:** ^1^ Faculty of Psychology and Cognitive Science, Adam Mickiewicz University, Poznan, Poland; ^2^ Cognitive Neuroscience Center, Adam Mickiewicz University, Poznan, Poland; ^3^ Human-Technology Interaction Group, Eindhoven University of Technology, Eindhoven, The Netherlands; ^4^ Department of Psychology, Stanford University, Stanford, CA, USA; ^5^ Department of Psychology, University of Rochester, Rochester, NY, USA

**Keywords:** affect, biopsychosocial, stress appraisals, reappraisal, challenge and threat, mindset

## Abstract

Affective responses during stressful, high-stakes situations can play an important role in shaping performance. For example, feeling shaky and nervous at a job interview can undermine performance, whereas feeling excited during that same interview can optimize performance. Thus, affect regulation—the way people influence their affective responses—might play a key role in determining high-stakes outcomes. To test this idea, we adapted a synergistic mindsets intervention (SMI) (Yeager *et al*. 2022 *Nature* **607**, 512–520 (doi:10.1038/s41586-022-04907-7)) to a high-stakes esports context. Our approach was motivated by the idea that (i) mindsets both about situations and one’s stress responses to situations can be shaped to help optimize stress responses, and (ii) challenge versus threat stress responses will be associated with improved outcomes. After a baseline performance task, we randomly assigned gamers (*n* = 300) either to SMI or a control condition in which they learned brain facts. After two weeks of daily gaming, gamers competed in a cash-prize tournament. We measured affective experiences before the matches and cardiovascular responses before and throughout the matches. Contrary to predictions, gamers did not experience negative affect (including feeling stressed), thus limiting the capacity for the intervention to regulate physiological responses and optimize performance. Compared with the control participants, synergistic mindsets participants did not show greater challenge responses or improved performance outcomes. Though our adaptation of Yeager *et al*.’s SMI did not optimize esports performance, our findings point to important considerations regarding the suitability of an intervention such as this to different performance contexts of varying degrees of stressfulness.

## 1. Applying a synergistic mindsets intervention to an esports context

Stressful, high-stakes performance situations are common in people’s lives, including school and university exams, public speaking, job interviews and sports competitions, to name just a few. These performance situations present acute task demands that require engagement—or orienting to demands—and instrumental responding to address these demands [1,2]. While some individuals thrive in such stressful performance settings and thereby attain goals, learn new skills or innovate outside their comfort zones, others wilt under pressure and fail to achieve their goals and thus stagnate. Findings from different research traditions indicate that two critical factors which shape how people perform in acutely stressful performance contexts are (i) how they evaluate—or *appraise*—certain aspects of the situations, and (ii) how they appraise their affective responses to those situations [2–9].

People tend to perform worse than might be expected when they appraise the situation (e.g. performance) as a threat—a belief that situational demands (e.g. controllability, social expectations and required effort) exceed a person’s resources (e.g. skills, knowledge and abilities; see [6] for a review). People also tend to perform worse than might be expected when they evaluate their physiological responses to the situation (e.g. sweaty hands) as harmful and hindering optimal performance [2]. The disadvantage of having these negative appraisals of either one’s situation and/or one’s responses to the situation (as opposed to more positive ones) has been shown in various performance contexts, including math tasks [10–12], surgery [13], flight simulation [14], darts [15], golf [16,17], gaming [18] and esports [19]. Understanding how these appraisals influence performance is a critical research goal that will assist in creating interventions to optimize individuals’ performance across a wide range of high-stakes situations.

### 1.1. How appraisals influence performance

**Figure 1.**
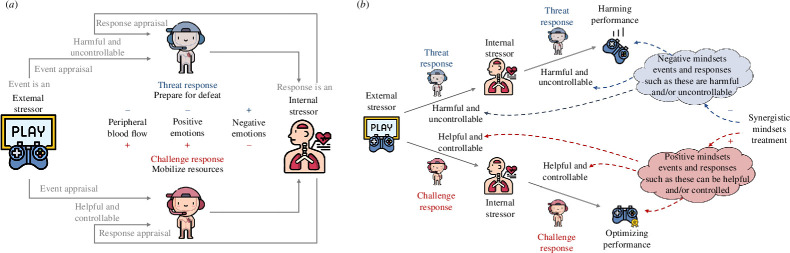
Impact of (*a*) appraisals and (*b*) reappraisals associated with the SMI during high-stakes performance situations. Figure adapted from [9]. Creative Commons Attribution (CC BY) license.

One possible mechanism by which appraisals of one’s situation and/or one’s responses to the situation can either harm or optimize performance outcomes is through the activation of one of two kinds of affective response: threat or challenge ([9]; figure 1).

When people have negative performance-related appraisals (e.g. the performance situation is perceived as harmful and uncontrollable), they tend to respond to the performance situation with the threat affective response [1]. Threat/challenge affective responses are operationalized via two loosely coupled responses: cardiovascular responses and affective self-reports. Threat affective response is associated with maladaptive cardiovascular responses, characterized by decreased cardiovascular efficiency—decreased blood flow throughout the body (i.e. decreased cardiac output and increased total peripheral resistance)—harming cardiovascular mobilization required for successful performance [3,20]. Cardiac output (CO) represents the volume of blood pumped through the cardiovascular system over time—usually 1 min (e.g. [21]). Decreases in CO indicates less cardiac efficiency and accompany avoidance-oriented threat states as less oxygenated blood is being delivered to the brain compared with states with higher levels of CO such as challenge. Total peripheral resistance (TPR) assesses overall vascular resistance. Increases in TPR suggest a reduction of blood flow to peripheral sites (i.e. concentrating blood in the core) and accompany threat states as the organisms prepare for damage or social defeat. Furthermore, threat responses are associated with increased negative affective experience and decreased positive affective experience—the affective profile that can be maladaptive [9] and that fits the worst emotional state for performance reported by athletes, namely feeling downhearted, sluggish and highly anxious [22]. Consistent with this idea, experiencing negative emotions such as anxiety and embarrassment is related to unsuccessful performance [23–25].

By contrast, when people have positive performance-related appraisals (e.g. the performance situation is perceived as helpful and controllable), they tend to respond to the performance situation with the challenge affective response [1]. Challenge affective response is characterized by vasodilation (i.e. reduced TPR) and increased blood flow throughout the body, which provides efficient energy expenditure and oxygenated blood to the brain and peripheral sites [3,20,26]. A previous meta-analysis shows that stronger challenge—rather than threat—cardiovascular response is related to better performance across multiple contexts (e.g. sports, education and cognitive tasks [26]). Furthermore, challenge responses are associated with increased positive affective experience and decreased negative affective experience [9]. This affective profile fits the ideal emotional state for performance reported by athletes, namely the mix of feeling happy, calm and energetic [22]. Consistent with this idea, researchers have found that upregulating positive emotions facilitates cycling performance [27], eliciting enthusiasm and amusement promotes better esports performance [19] and eliciting happiness promotes better sprint performance [28], strength performance and vertical jumping performance [29], and that experiencing positive emotions such as excitement and happiness is related to successful performance [23,25].

One note on terminology may be useful here. Although affective responses—stress responses and emotions—are often viewed as separate phenomena, they both involve appraisals and whole-body reactions to psychologically relevant situations [3,30–33]. We refer to affective responses and affect regulation—an umbrella term encompassing both coping and emotion regulation, among others [33]—as we believe they are the key to optimizing performance.

### 1.2. Sculpting appraisals using reappraisal

If appraisals of one’s situation and one’s physiological responses to that situation determine threat versus challenge affective responses [3,9,20], and if these affective responses help to determine performance outcomes, one obvious target for intervention is changing the negative appraisals of situation and response. The affect regulation studies in the performance domain that focused on intentionally changing the appraisals—a process known as reappraisal—can be divided into two groups.

The first group of reappraisal studies focuses on reappraisals of the situations [17,34–36;37] including viewing the situation as an opportunity to gain financial benefits with no negative consequences [34,37] , as a performance in which participants are doing very well [34], as a challenge to be met and overcome [17,34,35], as similar to previous performances [36], as a situation that lacks complications [36] and as a context, in which previous participants performed well [17,34,35]. Findings to date suggest that situation reappraisal is useful in shaping the challenge versus threat cardiovascular responses [17,28,35–37]. The studies have also provided initial evidence that situation reappraisals can help optimize performance outcomes, including motor [17,35,36] and cognitive tasks [38–40].

The second group of reappraisal studies focuses on individuals’ response reappraisals, including bodily sensations (e.g. arousal) and physiological responses [2,4,5,7,41]. An example of an intervention focusing on people’s responses is the stress-can-be-enhancing versus debilitating mindsets intervention [2,4,5]. The stress-can-be-enhancing intervention is based on the belief that psychophysiological stress responses (e.g. fast-beating heart or sweaty hands) are normal body reactions that mobilize energetic resources to provide optimal support for future actions. In that way, the body provides more blood with oxygen and energetic substances to the brain and the muscles [7]. Response reappraisal shifts the affective response, leading to greater challenge cardiovascular responses [7,42–46], more positive affect [47] and a more positive view of one’s physiological arousal and anxiety [48]. Response reappraisal has also been found to enhance performance across a wide variety of domains like motor tasks [16,49], academic exams [50], maths [43,51,52], artistic performance [43] and public speaking [42,43].

Although promising, the studies using reappraisal interventions (versus control conditions) show that effect sizes are modest and heterogeneous for both affective responses (ranging from *d* = 0.06 to *d* = 0.81 [21,53]; and for performance (ranging from *d* = −0.12 to *d* = 0.93 [16,54]; *d* = 0.10 in esports performance [34]). Furthermore, sometimes the effects are mixed, including non-significant effects of reappraisal intervention (e.g. [21,45,53,54]), mixed results for the same instructions and golf performance in different studies (*d* = 0.93 [16]; *d* = 0.00 [46] or significant results for a cardiovascular response, but not for affective experience [21,36]. These observations motivated us to use a novel double-barrelled approach called the synergistic mindsets intervention (SMI) that targets both situational and bodily reappraisals to achieve the best results [9].

### 1.3. Optimizing performance with the synergistic mindsets intervention

Mindsets are constellations of beliefs, which are temporally stable appraisals [55]. Recently, a new SMI has been proposed and empirically tested [9]. This SMI draws on the cumulative benefits of interventions related to reappraising situations (e.g. growth mindsets interventions [56,57]); and to reappraising responses to the situations (e.g. stress-can-be-enhancing interventions [2,7]). The SMI focuses on reappraising the performance situation as an opportunity to grow, show individuals' capabilities, flourish and strengthen the brain and on reappraising the bodily responses usually associated with stress as natural responses that provide optimal support for future actions (figure 1).

Another note on terminology may be useful. The SMI employed here builds on reappraisal interventions. The difference between targeted reappraisal interventions and more broadly focused mindset interventions lies in the scope of their impact, with the mindset intervention targeting general-level temporally stable beliefs and reappraisal interventions targeting situation-specific appraisals [9]. We chose this focus because (i) we believed that if it is possible to use reappraisal to change general beliefs (e.g. sweaty hands in various high-stakes situations are a normal bodily response), this should lead people to have challenge versus threat affective responses across many different contexts (see figure 1); and (ii) targeted reappraisal interventions may exhibit ‘transfer problems’ [58], whereby they may not generalize beyond the context in which the reappraisal is applied.

Yeager *et al*. [9] present six studies (total *n* = 4291) in which the SMI facilitated adolescents' stress-related cognitions to anticipated and experienced timed assignments, cardiovascular reactivity to a stressful task, daily cortisol levels, psychological well-being, academic success and anxiety symptoms during the 2020 COVID-19 lockdowns. Importantly, a synergistic approach that combined both types of reappraisals was associated with superior outcomes compared to interventions that focused on just one of the two types of reappraisals [9]. This premise is important for real-life applications where researchers strive to use and validate the best possible strategies while minimizing the costs of testing different available options (e.g., full factorial study designs).

Although previous studies in the affect regulation literature (including initial studies on the SMI) have many strengths, they also have some limitations that we aim to address in the present study. Previous studies usually focused on either emotional (e.g. [59–61]) or stress responses (e.g. [17,34,35,37]) rather than integrating them as affective responses. In both traditions (stress and emotions), interventions have focused on reappraising situations (e.g. [17,34–37]); or the responses to the situations (e.g. [16,21,42,43]), rather than focusing on both aspects of the performance. Studies generally have used brief and focal (i.e. single appraisal-oriented) reappraisal interventions rather than broader mindset-oriented interventions.

Although the initial studies on the SMI addressed these limitations [9], these studies, had different limitations that are worth noting, including a lack of multi-stage performance under controlled conditions with continuous real-time monitoring of physiological responses at multiple levels concurrently with performance. Thus, in testing optimal approaches for performers, we adapted and validated the SMI in real-world performance and examined its further potential in optimizing performers’ actions and affective responses.

### 1.4. Present study

The present research aimed to test the impact of SMI in a high-stakes context. In particular, we focused on esports, a relatively novel social phenomenon and the fastest-growing area in sports, in which well-trained individuals—gamers—compete using video games. In esports, gamers compete in the seated position in front of the screen, which provides an excellent opportunity to examine affective responses, namely affective experience and real-time cardiovascular responses related to performance in laboratory settings [19,34]. Using esports allowed us to examine high-stakes performance with continuous real-time monitoring of affective responses at multiple levels concurrently and answered the initial study authors' call for new large-scale trials in diverse populations and contexts [9].

We organized a large-scale study built around a real-life esports competition [19,34]. We conducted a three-stage experiment in which we introduced an intervention that was learned during a laboratory visit and practised in daily training. In stage 1 of the experiment (laboratory visit 1)—after a baseline performance match—half of the gamers were randomly assigned to SMI. The other participants were assigned to a control intervention focused on learning about the brain [9]. Next, participants were asked to apply the knowledge provided during the intervention in training performance matches. In stage 2, we tracked the daily training progress for gamers' affective experience and performance for two weeks. In stage 3, gamers were asked to compete in a cash-prize esports tournament (laboratory visit 2). We measured gamers' functioning in the laboratory visits with continuous, non-invasive measurements of affective responses (stages 1 and 3). Finally, we explored the long-term effects of the SMI with a one month follow-up.

We aimed to test the causal effect of the SMI and designed the study in a way that would allow us to examine the mechanism through which the causal effects operate. Relative to control condition gamers, we hypothesized that synergistic mindsets participants would (i) experience more positive and less negative affective experiences before the performance (hypotheses 1a and 1b; table 1 provides further detail about this and each subsequent hypothesis); (ii) display greater challenge cardiovascular response before the performance (hypothesis 2); and (iii) achieve better outcomes in the tournament performances (hypothesis 3) than control participants. We also expected that affective response (i.e. affective experiences and cardiovascular challenge response) would mediate the effects of the SMI on performance levels (hypotheses 4a, 4b and 5). In summary, our study provided a unique combination of internal and external validity (i.e. using a controlled experiment and real-world outcome), a robust assessment of affective and physiological dynamics and a robust theoretically motivated intervention.

**Table 1.** Design table.

question	hypothesis	sampling plan (e.g. power analysis)	analysis plan	rationale for deciding the sensitivity of the test for confirming or disconfirming the hypothesis	interpretation given to different outcomes	theory that could be shown wrong by the outcomes	results	
will SMI (versus control) lead to an increase in positive affective experience before the tournament performance in stage 3?	SMI (versus control) will lead to more positive affective experience before the tournament performance in stage 3 (hypothesis 1a)	prior works suggest an effect size of *d* = 0.45 for the difference between reappraisal interventions and the control conditions for hypothesis 1a. Our power simulation suggests that 250 participants (each playing eight matches) would provide over 95% power to detect the significant regression coefficient of *β* = 0.22 between the dummy-coded intervention group and positive affective experience	we will include the positive affective experience in the two-level SEM model as the outcome (latent factors). In the model, the intervention type will be introduced as a predictor (dummy-coded). We will account for the non-independence of observations by nesting each round of responses within individuals	we determined the relevant effect size for statistical power analysis based on effect sizes found in studies that tested similar research questions (see §2.2 for details)	if the positive affective experience is significantly higher (lower) in the synergistic mindsets conditions than in the control conditions, we will conclude finding evidence for (against) hypothesis 1a. If hypothesis 1a will be rejected, we will then use the equivalence test to determine whether synergistic mindsets and control intervention had the same effects on gamers. If the observed effect will lie inside the boundaries of the smallest effect of interest (–0.22, 0.22) and the CI around the observed effect does not overlap with the smallest effect of interest, we will conclude that the synergistic mindsets and control intervention has the same effects on gamers. If hypothesis 1a will not be rejected, we will then consider the practical value of the SMIs with minimum effect test. If the CI around the observed effect does not overlap with the smallest effect of interest (*r* = 0.22), we will conclude that the synergistic mindsets approach is a beneficial (harmful) approach for gamers	mainly: the synergistic mindsets model [9]; partially: the biopsychosocial model of challenge and threat [3], the growth mindset model [56,57], the arousal reappraisal model [2], the stress-can-be-enhancing mindset model [4,5]; in case of mixed findings (not significant and not equivalent), we do not draw full theoretical implications for alternative or null hypotheses	the SMI (versus control) did not lead to more positive affective experience before the tournament performance	
will SMI (versus control) lead to a decrease in negative affective experience before the tournament performance in stage 3?	SMI (versus control) will lead to less negative affective experience before the tournament performance in stage 3 (hypothesis 1b)	prior works suggest an effect size of *d* = 0.45 for the difference between reappraisal interventions and the control conditions for hypothesis 1b. Our power simulation suggests that 250 participants (each playing eight matches) would provide over 95% power to detect the significant regression coefficient of *β* = 0.22 between the dummy-coded intervention group and negative affective experience	we will include the negative affective experience in the two-level SEM model as the outcome (latent factors). In the model, the intervention type will be introduced as a predictor (dummy-coded). We will account for the non-independence of observations by nesting each round of responses within individuals	we determined the relevant effect size for statistical power analysis based on effect sizes found in studies that tested similar research questions (see §2.2 for details)	if the negative affective experience is significantly lower (higher) in the synergistic mindsets conditions than in the control conditions, we will conclude finding evidence for (against) hypothesis 1b. If hypothesis 1b will be rejected, we will then use the equivalence test to determine whether synergistic mindsets and control intervention had the same effects on gamers. If the observed effect will lie inside the boundaries of the smallest effect of interest (–0.16, 0.16) and the CI around the observed effect does not overlap with the smallest effect of interest, we will conclude that the synergistic mindsets and control intervention have the same effects on gamers. If hypothesis 1b will not be rejected, we will then consider the practical value of the SMIs with a minimum effect test. If the CI around the observed effect does not overlap with the smallest effect of interest (*r* = 0.16), we will conclude that the synergistic mindsets approach is a beneficial (harmful) approach for gamers	mainly: the synergistic mindsets model [9]; partially: the biopsychosocial model of challenge and threat [3], the growth mindset model [56,57], the arousal reappraisal model [2], the stress-can-be-enhancing mindset model [4,5]; in case of mixed findings (not significant and not equivalent), we do not draw full theoretical implications for alternative or null hypotheses	the SMI (versus control) did not lead to less negative affective experience before the tournament performance	
will SMI (versus control) lead to an increase in challenge cardiovascular before the tournament performance in stage 3?	SMI (versus control) will lead to greater challenge cardiovascular before the tournament performance in stage 3 (hypothesis 2)	prior works suggest an effect size of *d* = 0.44 for the difference between reappraisal interventions and the control conditions for hypothesis 2. Our power simulation suggests that 250 participants (each playing eight matches) would provide over 95% power to detect the significant regression coefficient of β = 0.22 between the dummy-coded intervention group and challenge cardiovascular response	we will include challenge cardiovascular response in the two-level SEM model as the outcome. In the model, the intervention type will be introduced as a predictor (dummy-coded). We will account for the non-independence of observations by nesting each round of responses within individuals	we determined the relevant effect size for statistical power analysis based on effect sizes found in studies that tested similar research questions (see §2.2 for details)	if the challenge cardiovascular response is significantly higher (lower) in the synergistic mindsets conditions than in the control conditions, we will conclude finding evidence for (against) hypothesis 2. This will lead us to the interpretation that using synergistic mindsets in performance may be a beneficial (harmful) strategy for gamers' health	mainly: the synergistic mindsets model [9]; partially: the biopsychosocial model of challenge and threat [3], the growth mindset model [56,57], the arousal reappraisal model [2], the stress-can-be-enhancing mindset model [4,5]; in case of mixed findings (not significant and not equivalent), we do not draw full theoretical implications for alternative or null hypotheses	the SMI (versus control) did not lead to greater challenge cardiovascular before the tournament performance	
will SMI (versus control) lead to better performance levels during the tournament in stage 3?	SMI (versus control) will lead to better performance levels during the tournament in stage 3 (hypothesis 3)	prior works suggest an effect size of *d* = 0.66 for the difference between reappraisal interventions and the control conditions for hypothesis 3. Our power simulation suggests that 250 participants (each playing eight matches) would provide over 95% power to detect the significant regression coefficient of *β* = 0.22 between the dummy-coded intervention group and performance level	we will include the performance measures in the two-level SEM model as the outcome. In the model, the intervention type will be introduced as a predictor (dummy-coded). We will account for the non-independence of observations by nesting each round of responses within individuals	we determined the relevant effect size for statistical power analysis based on effect sizes found in studies that tested similar research questions (see, §2.2 for details)	if the performance level is significantly higher (lower) in the synergistic mindsets than in the control conditions, we will conclude finding evidence for (against) hypothesis 3. If hypothesis 3 is rejected, we will then use the equivalence test to determine whether synergistic mindsets and control intervention had the same effects on gamers. If the observed effect will lie inside the boundaries of the smallest effect of interest (−0.03, 0.03) and the CI around the observed effect does not overlap with the smallest effect of interest, we will conclude that the synergistic mindsets and control intervention have the same effects on gamers. If hypothesis 3 will not be rejected, we will then consider the practical value of the SMI with a minimum effect test. If the CI around the observed effect does not overlap with the smallest effect of interest, we will conclude that the synergistic mindsets approach is a beneficial (harmful) approach for gamers	mainly: the synergistic mindsets model [9]; partially: the biopsychosocial model of challenge and threat [3], the growth mindset model [56,57], the arousal reappraisal model [2], the stress-can-be-enhancing mindset model [4,5]; in case of mixed findings (not significant and not equivalent), we do not draw full theoretical implications for alternative or null hypotheses	the SMI (versus control) did not lead to better performance levels during the tournament	
mediational hypothesis								
will the effects of SMI (versus control) on better performance levels be mediated by more positive affective experience before tournament performance in stage 3	effects of SMI (versus control) on better performance levels will be mediated by more positive affective experience before tournament performance in stage 3 (hypothesis 4a)	prior works suggest an effect size of *r* = 0.15 for the relation between positive affective experience and performance levels. Our power simulation suggests that 250 participants (each playing eight matches) would provide over 95% power to detect the significant regression coefficient of *β* = 0.15 between positive affective experience and performance levels. Furthermore, our power simulation suggests that 250 participants (each playing eight matches) would provide over 95% power to detect the total effect of reappraisal intervention (versus control) of *β* = 0.31 on performance levels, as well as the indirect effect of reappraisal intervention (versus control) of *β* = 0.03 on performance levels via more positive affective experience	in the two-level SEM model, we will include intervention type as a predictor (dummy-coded), the positive affective experience as the mediator (latent factor), and performance level as the outcome. We will account for the non-independence of observations by nesting each round of responses within individuals. We will test mediational effects because the inclusion of mediators often increases power relative to testing total effects only ([62,63]. Thus, testing mediations decreases the odds of type II error when less pronounced effects are studied	we determined the relevant effect size for statistical power analysis based on effect sizes found in studies that tested similar research questions (see §2.2 for details)	if the performance level is significantly higher (lower) in the synergistic mindsets conditions than in the control conditions owing to increased positive affective experience, we will conclude finding evidence for (against) hypothesis 4a. This will lead us to the interpretation that using synergistic mindsets in performance may be a beneficial (harmful) strategy for gamers' effectiveness, thanks to increased positive affective experience	mainly: the synergistic mindsets model [9]; partially: the biopsychosocial model of challenge and threat [3], the growth mindset model [56,57], the arousal reappraisal model [2], the stress-can-be-enhancing mindset model [4,5]; in case of mixed findings (not significant and not equivalent), we do not draw full theoretical implications for alternative or null hypotheses	effects of SMI (versus control) on better performance levels were not mediated by more positive affective experience. Furthermore, the way participants felt before matches did not influence the performance results	
will the effects of SMI (versus control) on better performance levels be mediated by less negative affective experience before tournament performance in stage 3?	effects of SMI (versus control) on better performance levels will be mediated by less negative affective experience before tournament performance in stage 3 (hypothesis 4b)	prior works suggest an effect size of *r* = −0.15 for the relation between negative affective experience and performance levels. Our power simulation suggests that 250 participants (each playing eight matches) would provide over 95% power to detect the significant regression coefficient of *β* = −0.15 between negative affective experience and performance levels. Furthermore, our power simulation suggests that 250 participants (each playing eight matches) would provide over 95% power to detect the total effect of reappraisal intervention (versus control) of *β* = 0.31 on performance levels, as well as the indirect effect of reappraisal intervention (versus control) of *β* = 0.04 on performance levels via less negative affective experience	in the two-level SEM model, we will include the intervention type as a predictor (dummy-coded), the negative affective experience as the mediator (latent factor), and performance level as the outcome. We will account for the non-independence of observations by nesting each round of responses within individuals. We will test mediational effects because the inclusion of mediators often increases power relative to testing total effects only [62,64 ]. Thus, testing mediations decreases the odds of type II error when less pronounced effects are studied	we determined the relevant effect size for statistical power analysis based on effect sizes found in studies that tested similar research questions (see §2.2 for details)	if the performance level is significantly higher (lower) in the synergistic mindsets conditions than in the control conditions owing to decreased negative affective experience, we will conclude finding evidence for (against) hypothesis 4b. This will lead us to the interpretation that using synergistic mindsets in performance may be a beneficial (harmful) strategy for gamers' effectiveness, thanks to decreased negative affective experience	mainly: the synergistic mindsets model [9]; partially: the biopsychosocial model of challenge and threat [3], the growth mindset model [56,57], the arousal reappraisal model [2], the stress-can-be-enhancing mindset model [4,5]; in case of mixed findings (not significant and not equivalent), we do not draw full theoretical implications for alternative or null hypotheses	effects of SMI (versus control) on better performance levels were not mediated by less negative affective. Furthermore, the way participants felt before matches did not influence the performance results	
will the SMI (versus control) on better performance levels be mediated by greater challenge cardiovascular response before tournament performance in stage 3?	effects of SMI (versus control) on better performance levels will be mediated by greater challenge cardiovascular response before tournament performance in stage 3 (hypothesis 5)	prior works suggest an effect size of *r* = −0.10 for the relation between challenge cardiovascular response and performance levels. Our power simulation suggests that 250 participants (each playing eight matches) would provide over 95% power to detect the significant regression coefficient of β = −0.10 between challenge cardiovascular response and the performance levels. Furthermore, our power simulation suggests that 250 participants (each playing eight matches) would provide over 95% power to detect the total effect of reappraisal intervention (versus control) of *β* = 0.31 on performance levels, 88% power to detect the indirect effect of reappraisal intervention (versus control) of *β* = 0.02 on performance levels via greater challenge cardiovascular response	in the two-level SEM model, we will include the intervention type as a predictor (dummy-coded), the challenge cardiovascular response as the mediator, and the performance level as the outcome. We will account for the non-independence of observations by nesting each round of responses within individuals. We will test mediational effects because the inclusion of mediators often increases power relative to testing total effects only ([62]; O'Rourke & MacKinnon, 2015). Thus, testing mediations decreases the odds of type II error when less pronounced effects are studied	we determined the relevant effect size for statistical power analysis based on effect sizes found in studies that tested similar research questions (see §2.2 for details)	if the performance level is significantly higher (lower) in the synergistic mindsets conditions than in the control conditions owing to greater challenge cardiovascular response, we will conclude finding evidence for (against) hypothesis 5. This will lead us to the interpretation that using synergistic mindsets in performance may be a beneficial (harmful) strategy for gamers' effectiveness, thanks to challenge cardiovascular responses	mainly: the synergistic mindsets model [9]; partially: the biopsychosocial model of challenge and threat [3], the growth mindset model [56,57], the arousal reappraisal model [2], the stress-can-be-enhancing mindset model [4,5]; in case of mixed findings (not significant and not equivalent), we do not draw full theoretical implications for alternative or null hypotheses	effects of SMI (versus control) on better performance levels were not mediated by greater challenge cardiovascular response. Furthermore, participants that displayed decreased cardiovascular challenge (stronger TPR increases) performed better	

## 2. Methods

### 2.1. Participants

Participants were 300 male gamers between the ages of 18 and 32 years (*M* = 21.95, s.d. = 2.29). Of the participants, 200 (67%) had not competed before, 76 (25%) had competed at the local level, 17 (6%) had competed at the national level, six (2%) had competed in international level tournaments and one did not respond. For 17 participants, esports activity provided additional income, whereas the rest did not make money from gaming. On average, participants had 9.13 years of experience in playing Counter-Strike: Global Offensive (CS: GO; s.d. = 5.22) and reported 2225.69 h of CS: GO gameplay (s.d. = 1980.55; Steam Library; Valve Corp., SA). Participants’ CS: GO ranks are reported in the electronic supplementary material, table S2. Of the participants, 300 completed stage 1, 298 completed stage 3 and 266 completed the follow-up questionnaires. We invited adult, Polish-speaking male players of one of the most popular esports games, CS: GO, who played at least 6 h per week. This criterion allowed us to collect the targeted sample, limiting it to experienced gamers [19,34]. Furthermore, we recruited adult participants because CS: GO is recommended only for +18 players [65]. We recruited Polish-speaking participants as the study was run in Poland. We recruited only male players owing to their predominance (76%) among first-person shooter gamers [66]. Including non-Polish and non-male participants would entail producing and testing different group-specific research materials. Furthermore, gender and language might become confounding factors to the study, which we could not test adequately owing to the expected small number of eligible participants from these groups. We recruited participants from the general population via a Facebook advertisement targeted at CS: GO gamers and mailing lists among university students in Poznan. In recruitment, potential participants were informed about the opportunity to participate in scientific research examining the psychological factors influencing esport performance. They were informed that the research would involve participating in the esports tournament with the main prize of 2500 PLN (*ca* $600) and that each participant would receive a 400 PLN (*ca* $100) shopping voucher. Participants provided informed consent.

### 2.2. Sampling plan

#### 2.2.1. Expected effect sizes

Since the initial study on SMI [9] did not test all associations included in our statistical model, we also considered other affect regulation studies to estimate the expected effect sizes. We used Cohen’s *d* and Pearson’s correlation *r* as effect size measures. In two cases [16,46], we calculated Cohen’s *d* from the results reported in the original study [67].

##### 2.2.1.1. Synergistic mindsets and performance

We found two studies examining the effects of similar interventions based on stress reappraisal in the sport performance domain: golf [16] and darts [46]. Previous reappraisal interventions found medium to large effect sizes (*d* = 0.70 [16]; *d* = 0.66 [46]), for the effects of reappraisal on performance.

Furthermore, we calculated the smallest effect size of interest to interpret the effect sizes regarding the statistical significance and practical significance [68]. We decided that the smallest effect size of interest for performance outcome in our study should mimic the difference between the rivals ranked three places apart in the tournament table. Based on the data from our previous study, where we measured esports performance [34], we calculated the effect size for the difference (e.g. the difference between 1st and 4th places, or 7th and 10th place). We found a very small effect size (*d* = 0.07, 95% confidence interval (CI) = [−0.24, 0.38]) for rivals ranked three places apart. Thus, the smallest effect size of interest for performance outcome in our study would be *d* = 0.07.

For the associations between performance level and SMI, both types of effect sizes seem impractical for our project’s power analysis. The effect sizes in the literature are surprisingly large, as affect reappraisals usually have smaller effects on affective experience [69,70] than the observed effects on the performance, which seems *a priori* unlikely, given that the effect on performance is theoretically expected to be mediated by the effect on the affective experience. Thus, we are sceptical that the expectations based on effect sizes in the literature that affect reappraisal should have stronger effects on the performance levels than on the affective experience are realistic.

By contrast, aiming to achieve power for finding the smallest effect size of interest would require huge resources, and although designing a study to detect or reject the smallest effect size of interest would be most informative, given the substantial uncertainty in the presence of effects and their size, we believe a first important step is to examine the presence or absence of effects in a more realistic range. In the result of weighting between what can be done and what is expected, we used a more conservative effect size than the expected effect size based on published findings. We designed the study to be able to detect the effects for the association between the intervention and performance outcomes of *d* = 0.45. Thus, our approach would be able to really improve the accuracy of effect sizes in the sports performance field, which is valuable even though tiny effects in sports might matter, those are too costly to detect now.

##### 2.2.1.2. Synergistic mindsets and affective experience

We used one meta-analysis [70] and one recent large-scale study [69] to estimate the possible effect sizes. Meta-analysis shows that reappraisal has an average effect size of *d* = 0.45, 95% CI = [0.35, 0.56] in changing affective experience relative to passive control conditions (i.e. no instruction, instructions to experience naturally, instructions to not regulate in a specific manner or instructions to enhance or maintain the focal emotion). A recent large-scale study found similar effects of reappraisal interventions (versus controls) on positive affective experience *d* = 0.59 and on negative affective experience *d* = 0.39 [69].

Based on the previous study, we estimated the smallest effect size of interest of reappraisal interventions on the increase of positive affective experience of Δ*d_z_* = 0.47 and decrease of negative affective experience of Δ*d_z_* = 0.32 [68].

##### 2.2.1.3. Synergistic mindsets and cardiovascular challenge/threat responses

We found three studies examining the effect of similar interventions based on stress reappraisal that measured physiological challenge/threat cardiovascular responses in the performance domain [9,16,46]. Previous reappraisal interventions showed medium to large effect sizes (*d* = 0.44 [16]; *d* = 0.90 [46]; *d* = 0.44–0.79 [9]), for the effects of reappraisal intervention of challenge/threat cardiovascular responses. We did not calculate the smallest effect size of interest for cardiovascular changes because we were unable to conceptualize the practically interesting change of cardiovascular measures.

##### 2.2.1.4. Affective experience and performance

The published correlation coefficients for associations between performance and experienced emotions and stress are inconclusive, namely *r* = 0.14 for anxiety [71], *r* = 0.10 for excitement [71], *r* = 0.10 for happiness [71], *r* = −0.09 for anxiety [72], *r* = −0.18 for excitement [72], *r* = −0.19 for happiness [72], *r* = 0.15 for positive affective experience [73] and *r* = −0.14 for negative affective experience [73]. The effects are stronger for associations between performance satisfaction and positive affective experience (*r* = 0.31 [74]; *r* = 0.52 [75]); and negative affective experience (*r* = −0.24 [74]; *r* = −0.36 [75]). However, they do not account for the objective performance results. Thus, to calculate the expected effect size, we used unpublished data [76] from our previous project that closely resembled a gaming tournament situation where we measured affective experience before and during gaming performance [34] and found small effect sizes for the associations between affective experience and performance levels (*r* = 0.15; *d* = 0.30 for positive affective experience and *r* = −0.15; *d* = −0.30 for negative affect).

##### 2.2.1.5. Cardiovascular challenge/threat responses and performance

Based on the meta-analysis, we expect small effect sizes for the effects of cardiovascular responses on performance levels (*r* = 0.10–0.14; *d* = 0.20–0.28 [26]).

### 2.2.2. Sample size determination

We calculated the required sample sizes for our study with the Monte Carlo simulations using Mplus 8.0 [77]. To determine the sample size, we considered expected effect sizes and the smallest effect size of interest [78]. To run the power analysis for the structural equation model, we transformed Cohen’s *d* into Pearson’s correlation *r* with an effect size converter [79]. In the simulation model, we used the following correlation coefficients: affect regulation group on performance *r* = 0.22 (based on conservative effect sizes in the literature), on positive affective experience *r* = 0.22, on negative affective experience *r* = −0.22 (based on effect sizes in the literature [69,70]), and on challenge/threat cardiovascular response *r* = 0.22 (based on effect sizes in the literature [9,16,46]), positive affective experience on performance *r* = 0.15; negative affective experience on performance *r* = –0.15 (based on analysis of unpublished data from [34]); challenge/threat cardiovascular response on performance *r* = 0.10 (based on effect sizes in the literature [26]). We used values of 0.50 for factor loadings for the positive and negative affect. We based our assumptions on the previous studies, which observed the factor loadings ranging from 0.53 to 0.79 for positive affective experience and from 0.47 to 0.65 for negative affective experience [80,81].

We found that for two-level mediational models (for a repeated measures design), detecting expected small-to-medium effect sizes with a power of at least 0.95 and α = 0.05 would require a sample size of 2000 cases—250 participants, each playing eight matches.

Type 1 and Type 2 errors are weighed equally. This stems from the balance between the costs and benefits of the synergetic mindset intervention. The computerized intervention is relatively cheap; thus, a Type 1 error is not costly. The potential benefits for gamers if the SMI optimizes the performance are relatively large. Thus, rather than using the default 1 : 4 ratio between Type 1 and Type 2 errors (*α* = 0.05, *β* = 0.20, power 0.80), we used a 1 : 1 ratio [82].

We also calculated the power for the model fit index of Root Mean Square Error of Approximation (RMSEA) [83]. We found that our model (*α* = 0.05; d.f. = 37; sample size = 2000; null RMSEA = 0.01; alt. RMSEA = 0.05) should have had a power of 1.00 to detect RMSEA of 0.05. We included the code for the power analysis in the electronic supplementary material.

In anticipation of potential data loss, additional participants were recruited (up to *n* = 300), expecting 10–20% of the sample to be reduced owing to physiological recording problems and voluntary attrition. We expect a reduction in the sample based on our experience with similar projects [19,34]. Based on these power calculations, we secured funding for 300 participants. Thus, data collection terminated after 300 participants were enrolled and provided data. The Monte Carlo simulation script is available in the electronic supplementary material. Furthermore, the sensitivity analysis showed that our sample allowed us to detect the effect sizes of *d* = 0.42 with *α* = 0.05 and *β* = 0.95 for the difference between the two independent groups for the secondary variables (e.g. negative prior mindsets).

### 2.2.3. Exclusion

We used participants’ preparation standards (e.g. refraining from physical exercise and intake of medications and caffeine for 2 h before testing) and exclusion criteria (e.g. significant cardiovascular health problems or diagnosed mental disorders) used in psychophysiological studies (e.g. [84]). Furthermore, we asked participants to reschedule if they experienced illness or a major adverse life event.

We also excluded participants identified as careless responders. We identified the participant as a careless responder if he: (i) selected other answers than ‘strongly agree’ for the item: ‘please select “strongly agree” for this item to show that you are paying attention’; (ii) answered the whole baseline questionnaire with a string of identical responses greater than 40 items (half the length of the total scale [85]); and (iii) answered the last item of the baseline questionnaires—‘in your honest opinion, should we use your data in our analyses in this study’—‘no, I responded carelessly’ [86].

We identified five participants as careless responders. They failed to complete the ‘directed query’ attention check (four in visit 1 and one in visit 2). We did not identify any participants as careless responders based on other criteria. We excluded them from the analysis. During recruitment, we excluded participants who met diagnostic thresholds for problematic gaming. To screen participants, we used the Gaming Disorder Test (GDT [87]; see the electronic supplementary material for details), a validated psychometric test [88,89] developed to assess gaming disorders defined in the International Classification of Diseases (ICD-11 [90]). The GDT was a part of the online study registration. We identified eight (of 885—1%—of volunteers who showed interest and filled in the recruitment form) who met diagnostic thresholds for problematic gaming (i.e. endorsement of all four diagnostic criteria as assessed by each GDT item: marking ‘4: often’or ‘5: very often’ [87]). Before inviting these participants, we referred them to a clinical psychologist. Only one volunteer visited the clinician and was judged to have a diagnosis of gaming disorder. Thus, none of the eight gamers who met diagnostic thresholds for problematic gaming were invited to participate in the study.

Although participants were scheduled on the same day for laboratory visits 1 and 2, two weeks apart, some participants were not able to visit the laboratory on the scheduled day. We did not exclude participants for whom stage 2 was longer than 14 days, as the amount of esports training was self-decided by the participants. We added esports training time as the moderator in the exploratory analysis, where we tested the robustness of our findings.

### 2.3. Procedure

**Figure 2.**
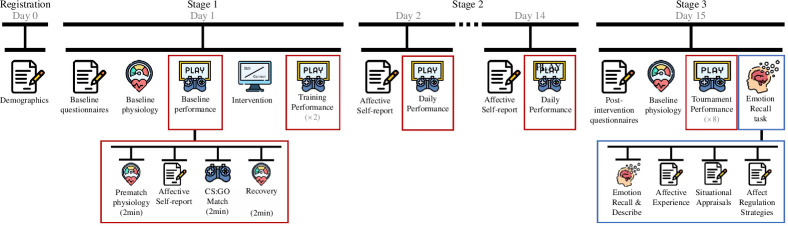
Project and match procedures.

Each participant visited the laboratory twice for individual testing (figure 2). During the first laboratory visit, we collected baseline and training measures (stage 1). Participants were randomly assigned to receive either the control instructions, which focused on brain facts, or the SMI, which focused on using reappraisal to think more productively both about the performance situation and their responses to this situation. Participants in the SMI condition continued reappraisal for two weeks and reported their daily adherence and progress (stage 2). During the second laboratory visit, participants competed in the esports tournament (stage 3). Finally, we explored the long-term effects of the SMI with a one month follow-up, where participants answered the same questionnaire set as at the beginning of stages 1 and 3.

#### 2.3.1. Stage 1

During the first laboratory visit, after arriving at the laboratory, participants were instructed on what they would be doing during the study. They provided informed consent, in which they could choose what data they wanted to make publicly available. Next, the researcher applied psychophysiological sensors, and participants filled in the baseline questionnaires, including measures of situational affect regulation, affective experience, negative prior mindsets, well-being and ill-being, alexithymia and emotion beliefs (for details, see §2.4 and the electronic supplementary material). The order of the baseline questionnaires was randomized to minimize the order effects. All instructions were presented, and responses were collected via two PCs with 23 in. screens (one for gaming and the second for experimental software). Once the participant completed the questionnaires and the experimenter switched on all the equipment and software, the experimenter left the room and the experiment began.

The experiment started with a 5 min physiological baseline (for the full descriptions of physiological measures, see §2.4), during which we asked participants to sit quietly. After baseline measures, participants were asked to play the first match—baseline performance (without manipulations). The baseline performance format resembled a future tournament. After the match, participants were randomly assigned to one of two conditions: the SMI or the control group. Participants were randomly assigned using a random number generator (www.randomlists.com/) (the randomization scheme is presented in the electronic supplementary material, table S3). Both groups completed the self-administered 30–40 min intervention.

The interventions were presented as part of an upcoming training programme for young gamers to prepare them for the demands of competitive esports. Thus, as part of the study, we asked participants to help us test one of the modules of the psychological training programme for the future generation of gamers. We informed the players that we were testing different modules that we planned to use in the training programme, but before including them, we needed to test which programme elements were beneficial to gamers and that, owing to logistics, we would present them only one of the modules (related to stress and emotions or related to the brain). After the interventions, participants were asked to evaluate the presented modules. Blinding was maintained by emphasizing to participants that each module was created to help them develop powerful new psychological skills and prepare them to accomplish their goals. Although the experimenters did not have information about the intervention assignment before the study, they gained it from the instructions displayed during the sessions.

Next, participants were asked to apply the knowledge they gained during the esports training performance (two matches). The training performance format also resembled a future tournament. At the end of stage 1, participants were instructed on how to report daily measures. Participants in the synergistic mindsets condition were instructed to use the affect regulation strategy during gaming as often as possible in the coming two weeks. Participants in the control group were encouraged to apply the information they learned to daily gaming.

##### 2.3.1.1. Synergistic mindsets intervention

We adapted the SMI [9] previously used in an educational context to the esports performance context. The intervention is based on two active ingredients.

The first ingredient aims to change the appraisals of the performance situation. It introduces the idea that stressful and unpleasant performance situations might be, in fact, an opportunity to show one’s capabilities and flourish, where one can manage how one feels. As with all challenging situations, first, gamers need to overcome many struggles, and they eventually get better with practise. Participants were asked to embrace challenges so they could grow their skills and learn how to regulate stress and emotions. The intervention makes a case for the possibility of improving over time based on neuroscientific information about the brain’s potential to develop more efficient (i.e. ‘stronger’) connections when people face difficult challenges and keep trying until they get better. To illustrate this idea, we used the analogy of muscles getting stronger with training [91]. The intervention aims to overcome the fixed mindset beliefs that often present intellectual and other abilities as fixed personal characteristics that cannot be changed [56]. A fixed mindset leads to negative appraisals about controllability, efforts, causes of failures and desired goals [56,57].

The second ingredient targets appraisals related to bodily responses to performance. Our intervention explained that when people engage in performance, they may experience body reactions such as a racing heart and sweaty palms—usually appraised as a harmful stress response. The intervention led people to perceive those body signals as information that the body is naturally preparing to provide optimal support for future actions and might be associated with positive emotions like excitement [2,4,5]. In that way, the body provides more blood with oxygen and energetic substances to the brain and the muscles [7]. Thus, the physiological response is proposed to be helpful for gamers. These appraisals align with a stress-can-be-enhancing mindset [4,5] in which stressors and stress responses are no longer valued as only ‘bad for me’ but perceived as being potentially ‘good for me’.

In the SMI, the two elements were presented synergistically, building on each other to form a coherent whole. People learned that by reappraising different stressors—situations and responses to those situations—they can build an effective response which can optimize their performance (figure 1). In the intervention, we not only informed the participants about scientific findings about mindsets but also guided them on how to regulate affective responses using reappraisal. The reappraisal was presented as targeting how one thinks about both the performance situation and the response to the situation [92] *e*. We encouraged participants to share their experiences about stress and emotions felt while gaming and their regulation strategies. Participants also heard stories from other players who shared their stories of using reappraisal in gaming. Participants were encouraged to share what they learned during the intervention as advice for someone else in a similar gaming situation, modelled after the ‘saying-is-believing’ writing exercise [93]. The detailed intervention instructions are presented in the electronic supplementary material.

##### 2.3.1.2. Control condition

Participants in the control condition learned basic scientific information about brain functioning. The intervention was a 20–30 min self-administered activity designed to be visually similar to the SMI. The content was modelled after the control condition used in prior large-scale growth mindset experiments [9,94] and created with the design-thinking method [95]. The module resembles a psycho-educational talk about the foundations of how the body and mind work, which is usually one of the first parts of mental training programmes for athletes when the term ‘physiological arousal’ is introduced [96,97]. Next, to mirror the activity in the synergistic mindsets group, participants were encouraged to share their feedback and read stories from other gamers that helped us adapt the interventions. Information in the control condition did not make any claims about mindsets, affect regulation skills or appraisals of the performance situation and responses to it.

##### 2.3.1.3. Gaming performance

Participants played CS: GO. In this multiplayer team-based first-person shooter game, two teams compete in simulated military combat. CS: GO is the leading game in the esports team-play category. It is also a popular leisure activity that engages up to 1.1 million daily active gamers worldwide [98]. In CS: GO, gamers compete online against other gamers or offline against computer-controlled characters. To standardize conditions across participants, each participant competed in a deathmatch mode on the Dust II map against computer-controlled avatars (bots) set at the maximum difficulty level (expert) without random weapons. Thus, we created a human–computer interaction situation (not human–human interaction). The game system calculated each match score by multiplying the points for eliminating each enemy bot by the weapon difficulty level. Higher scores indicate better performance [34]. All gaming matches consisted of prematch baseline measurements (2 min), gaming (2 min) and recovery (2 min) (figure 2). Throughout all match phases, we collected cardiovascular data. The participants reported their affective experience and provided demands and resource evaluations before each match.

### 2.3.2. Stage 2

Between laboratory visits, participants were asked to play CS: GO as frequently as they typically did. On days on which they chose to play CS: GO, we asked participants to select one of their gaming sessions that day to play in performance mode, as they would in a tournament. Participants were asked to play a single daily match in a mode resembling a future tournament, including a 2 min waiting period and affective self-reports before the match—daily performance (figure 2). After the match, participants were asked to report match scores. At the end of the day, participants were asked to report their daily positive and negative affective experiences and how much time they played during the day.

During the first week of stage 2, participants in the SMI were asked before the match to use reappraisal to regulate their affective response. During the second week, they did not receive such information. This was to observe whether participants learned and used reappraisals in daily gaming without being asked directly. Furthermore, the synergistic mindsets group reported adherence and progress in scheduled affect regulation training. As in a similar study [99], for each daily entry, participants reported the affective gaming situations in which they applied the reappraisal to ensure compliance with affect regulation instructions. The instructions were: ‘list some of the gaming situations that elicited strong emotions or stress and the way you used rethinking to make the situation beneficial to you’. This question can be treated as a reminder or booster of the SMI, as participants were asked daily to describe the situation in which they applied the knowledge learned during the intervention. Participants in the control group were asked to: ‘list some of the gaming situations that happened to you today’.

As the frequency and optimal dose are essential for the effectiveness of interventions [100], we asked participants to train daily. Yet, participants were informed that they could adjust how often, how long to practise and at what difficulty level. This minimized the risk of boomerang effects or unintended counter-reactions.

### 2.3.3. Stage 3

During laboratory visit 2 (two weeks after laboratory visit 1), participants competed in the esport tournament. The session began like stage 1, with a physiological hook-up, a set of questionnaires, and a 5 min physiological baseline. Next, participants played eight tournament matches—tournament performance. After the tournament, participants were asked to report the negative appraisals and complete an emotion recall task. Finally, participants evaluated how they perceived the study using intervention evaluation measures. Upon completion, participants were debriefed, screened for suspicion and offered 400 PLN (*ca* $100) vouchers. Winners received 2500, 1500 and 1000 PLN for taking first, second and third places (*ca* $600, $360 and $240).

#### 2.3.3.1. Emotion recall task

Participants were asked to recall, describe and evaluate two tournament situations, one that elicited positive emotions and one that elicited negative emotions. Participants were asked to evaluate the situations on the dimensions of positive and negative affective experience, appraisals and affect regulation strategies.

### 2.4. Measures

We collected four types of measures in this project. First, we collected measures for manipulation checks, description and exploration of potential outcomes of the SMI, including intervention evaluation, situational affect regulation, demands and resource evaluations, negative appraisals, situational appraisals and demographics. We treated them as secondary because we did not include them in the power analysis, and we may not have enough statistical power to infer the effects of the SMI on them. Second, we collected primary measures related to the main research questions used in power analysis for the sample size determination, namely affective experience, challenge/threat cardiovascular responses and performance outcomes. Third, we collected measures that can serve as possible moderators of the effects of SMI, including negative prior mindsets, self-esteem, interoception abilities and gaming experience. Fourth, we collected measures outside the scope of this report (e.g. video recordings of participants and their gameplay, leg movements, well-being and ill-being). The measures outside this report’s scope and unrelated to the research questions are presented in detail in the electronic supplementary material. All data and materials are available in the repository on the Open Science Framework website.

#### 2.4.1. Manipulation check measures

##### 2.4.1.1. Intervention evaluation

We measured the intervention acceptability with the 7-item Program Feedback Scale (PFS [101]). The PFS includes items such as ‘I enjoyed the program’. Furthermore, we measured the motivation to apply the information included in the interventions and the belief in the effectiveness of the affect regulatory information included in the interventions [69]. We asked about the motivation to use information included in the interventions with the item: ‘I will try my hardest to apply information included in the program’. Belief in the effectiveness of information included in the interventions was measured with the item ‘I believe that using the information included in the program will facilitate my gaming performance’. Participants answered on a 7-point scale ranging from 1 (strongly disagree) to 7 (strongly agree). We used our Polish translation of the scale. The scale has been used in intervention-based studies (e.g. [101–103]) and showed good internal consistency (Cronbach’s *α* = 0.88 [101]). Our data supported scale acceptable internal consistency (Cronbach’s *α* = 0.84 in T1 and 0.87 in T2).

##### 2.4.1.2. Situational affect regulation

We measured situational affect regulation using items from the Regulation of Emotion Systems Survey–Ecological Momentary Assessment (RESS-EMA [104]). The RESS-EMA captures the use of emotion regulation strategy, including distraction, reappraisal, rumination, suppression, engagement and the relaxation subscales. From the original 12 items from the RESS-EMA, we only used six items: ‘I took deep breaths’ (relaxation); ‘I expressed my feelings’ (engagement); ‘I continually thought about what was bothering me’ (rumination); ‘I thought of other ways to interpret the situation’ (reappraisal); ‘I engaged in activities to distract myself’ (distraction); ‘I made an effort to hide my feelings’ (suppression). Participants answered on a 7-point scale ranging from 1 (strongly disagree) to 7 (strongly agree). We used our Polish translation of the scale. The 12-item scale has been used in affective research (e.g. [104–107]) and shows good internal consistency (subscales Cronbach’s *α* levels ranging from 0.88 through to 0.94 [105]). The validity of using half of the items from the original scale had not been tested prior to our study. Because we used the shortened version of the scale with only a single item for each affect regulation strategy, we did not calculate Cronbach’s *α* values.

##### 2.4.1.3. Situational appraisals

We measured participants' appraisals related to emotional situations using a 10-item appraisal scale [108]. The appraisal scale captures dimensions representing five core appraisals, namely, relevance for goals and motives, congruence with goals and motives, accountability, outlook certainty and coping potential. The appraisal scale includes items such as ‘… I had a sense that this situation mattered to me’ (relevance), ‘… I had a sense that this situation was potentially desirable for me’ (congruence), ‘… I had a sense that I was responsible for this situation’ (accountability), ‘… I had a sense that I did not know how this situation was going to turn out’ (outlook certainty), ‘… I had a sense that I could change this situation for the better’ (coping potential). Participants answered on a 7-point scale ranging from 1 (strongly disagree) to 7 (strongly agree). We used our Polish translation of the scale. The scale has been used only in the initial study [108]. The items were designed to measure independent appraisal dimensions, as shown in the initial study (a mean pairwise Pearson correlation of 0.14 within a range from 0.00 to 0.50 [108]; and our study (a mean pairwise Pearson correlation of 0.17 and 0.19 within a range from 0.00 to 0.58.

##### 2.4.1.4. Negative appraisals

We measured participants’ appraisals related to the tournament using four items related to demands and resources [9]. Participants rated their agreement or disagreement with the statements, including ‘today’s tournament felt like a negative threat to me’; ‘today’s tournament felt like a positive challenge to me’; ‘I felt like my body’s stress responses helped my performance in today’s tournament’; and ‘I felt like my body’s stress responses hurt my performance in today’s tournament’. Participants answered on a 5-point scale ranging from 1 (strongly disagree) to 5 (strongly agree). Data from our project showed slightly below the acceptable internal consistency level for the scale (Cronbach’s *α* = 0.67). The ratings (reversed for positive items) provide a situational appraisal index, with higher values corresponding to more negative appraisals. We used our Polish translation of the items. The items were used in the initial synergistic mindsets study [9].

##### 2.4.1.5. Demands and resource evaluations

We measured cognitive appraisals of situational demands and personal resources using an appraisal ratio approach [9,17,35,109]. One item assessed task demands (‘how demanding do you expect the CS: GO match to be?”), and another item assessed personal resources (‘how able are you to cope with the demands of the CS: GO match?”). The scale ranged from 1 (not at all) to 6 (extremely). A ratio was calculated by subtracting demands from resources (range: −5 to +5), with a more positive value reflecting a challenge state and a more negative value reflecting a threat state [17]. We used our Polish translation of the items.

##### 2.4.1.6. Demographics

Participants reported their performance level (the highest level of competition: recreational, local, national, international), professional level (esport as full-time job, part-time job, no-income activity), duration of weekly playing (in hours for a typical week), experience and in-game ranking and measured as the highest rank achieved in the last 12 months. We calculated how many hours participants played during the last two weeks based on their daily reports. Furthermore, participants reported their age, body mass index, and education.

### 2.4.2. Primary measures

#### 2.4.2.1. Affective experience

To measure emotions and stress, we asked participants how they felt at the end of the prematch baseline. We used items from the modified Differential Emotions Scale to assess emotions and stress participants felt ‘right now’ [110]. For positive affect, we measured four items: amusement, excitement, joy and pride. For negative affect, we measured four items: anger, fear, overwhelm and stress. Before the matches, the scales ranged from 1 (strongly disagree) to 7 (strongly agree). (All response options were labelled, and numbers were displayed to participants for clarity; details for all scoring rules are described in §2.5.1.) The data from our project for the scales showed good internal consistency (Cronbach’s *α* = 0.74–0.88). We used our Polish translation of the items. Similar measures were used in the previous studies and showed high internal reliability, ranging from Cronbach’s *α* = 0.82–0.94 (e.g. [69,111–113].

#### 2.4.2.2. Challenge/threat cardiovascular response

We collected cardiac biosignals using the Vrije Universiteit Ambulatory Monitoring System (VU-AMS; The Netherlands). VU-AMS includes impedance cardiography (ICG) and electrocardiography (ECG) that allow the recording of cardiac action continuously and noninvasively. Following psychophysiological guidelines [114,115], we used pre-gelled AgCl electrodes (Kendall Abro, H98SG) placed in a standard Lead II configuration for ECG and a four-spot electrode array for ICG. The recordings were processed using the VU-AMS Data, Analysis & Management Software (VU-DAMS 5.4.20). After detecting B, C, X and R points in the ECG and ICG, we (M.B. and P.C.) visually checked and adjusted all point markers when necessary to correct erroneous placements. Then, we calculated heart rate (HR; reported as number of beats per minute (bpm)) the pre-ejection period (PEP; the period from initiating ventricular depolarization to opening of the aortic valve and ejection of blood reported in milliseconds) and CO (the amount of blood pumped by the heart per minute reported in litres ) CO is calculated by first estimating stroke volume—the amount of blood ejected during each beat—and multiplying that by heart rate.

We collected cardiovascular biosignals using Finometer MIDI (Finapres Medical Systems, The Netherlands). Finometrer MIDI uses the volume-clamp method with finger cuffs to record finger arterial pressure waveforms. It allows us to estimate systolic blood pressure (reported in mmHg), diastolic blood pressure (reported in mmHg) and TPR (a measure of the total vascular resistance reported in mmHg·min l^-1^).

Responses along the cardiovascular challenge/threat dimension were operationalized as PEP, HR, CO and TPR responses. Shorter PEP reflects sympathetic activation [20]. Shorter PEP and higher HR are characteristics of task engagement and physiological readiness for motivated performance and are considered prerequisites for interpreting CO and TPR as physiological indicators of psychological challenge and threat [3]. This initial cardiovascular response leads to challenge- or threat-specific reactions. Challenge cardiovascular response is characterized by greater cardiac efficiency (i.e. increased CO) with lower vascular resistance (i.e. decreased TPR) than threat cardiovascular response [20]. TPR is a primary measure of challenge/threat cardiovascular response and measures of the resistance to blood flow in the circulatory system [3,9]. The TPR is determined by the resistance of the arterial and venous vessels, as well as by any changes in the diameter of the vessels owing to vasoconstriction or vasodilation. It affects the amount of blood flow and the pressure at which it flows through the body. Owing to the technical limitations—participants in our study used both hands during the game—we collected PEP, HR and CO but not TPR during the esports matches.

#### 2.4.2.3. Performance

We used the match score as the primary performance level indicator. The CS: GO game system calculates each match score by multiplying the points for eliminating each enemy bot by the weapon difficulty level. A higher score indicates better performance. Daily reports asked participants to report the match score simulating the laboratory gaming tournament. Furthermore, we collected secondary performance measures, including the number of kills, kills’ assists and deaths.

### 2.4.3. Moderators

#### 2.4.3.1. Negative prior mindsets

We assessed participants’ fixed and stress mindsets with the 3-item Growth Mindset Scale (GMS [55]; Polish adaptation—[116] and 3-items from Stress Mindset Measure (SMM [5]; Polish adaptation—[117]). The GMS includes items such as ‘your intelligence is something about you that you can't change very much,’ and the SMM includes items such as ‘the overall effect of stress on my life is negative’. Participants answer on a 5-point scale ranging from 1 (strongly disagree) to 5 (strongly agree). Data from our project indicated good internal consistency (GMS Cronbach’s *α* = 0.92–0.96, and SMM Cronbach’s *α* = 0.78–0.88). We used a Polish translation of the scales. These measures have been used in affective studies (e.g. [4,5,9,118,119]) and showed good internal consistency for GMS (Cronbach’s *α* = 0.90 [118]; between 0.70 and 0.85 [9]; and for SMM (Cronbach’s *α* = 0.80 [5]; Cronbach’s *α* = 0.91 [117]).

#### 2.4.3.2. Self-esteem

We measured participants’ self-esteem with the Single-Item Self-Esteem Scale [120]. Participants rated their agreement or disagreement with the statement ‘I have high self-esteem’ on a 7-point scale ranging from 1 (strongly disagree) to 7 (strongly agree). We used our Polish translation of the item. This measure has been used in previous affective studies (e.g. [121–123]).

#### 2.4.3.3. Interoception

We measured participants’ interoception abilities with the 18-item body awareness questionnaire [124]. Participants rated their agreement or disagreement with the statements, including ‘I notice distinct body reactions when I am fatigued’, on a 7-point scale ranging from 1 (strongly disagree) to 7 (strongly agree). Data from our project indicated good internal consistency (Cronbach’s *α* = 0.74–0.88). We used a Polish translation of the scale [125]. This scale has been used in the affective studies (e.g. [126,127]) and showed good internal consistency (Cronbach’s *α* = 0.80 [125]; Cronbach’s *α* = 0.82, [124,127]).

#### 2.4.3.4. Gaming experience

We used the total time spent playing the CS: GO that is counted CS: GO game system (Steam Library; Valve Corp., USA) as the gaming experience indicator. Before stage 1, we asked participants to report the total hours played. Participants also reported the duration of playing against bots (in hours for a typical week).

### 2.4.4. Response quality checks

We had a single ‘directed query’ attention check [128]. In questionnaire sets, the Emotion Beliefs Scale contained an additional item: ‘please select ‘strongly agree’ for this item to show that you are paying attention’. We also had a single attention and effort check [86]. At the end of the questionnaire sets, we asked participants: ‘in your honest opinion, should we use your data in our analyses in this study?.

### 2.5. Analysis plan

Researchers were blind to the group allocation while running data pre-processing and analysis.

#### 2.5.1. Data pre-processing

##### 2.5.1.1. Outliers

We identified outliers with the median absolute deviation, with a cut-off of 3, as recommended by Leys *et al*. [129,130]. We then deleted the data if the data were identified as an error. We did not observe any measurement errors or encoding errors in affective experience data or gaming data. For the cardiovascular data, we double-checked the identified outliers and deleted biologically impossible values.

##### 2.5.1.2. Missing data

After excluding five careless responders and deleting data identified as an error, our dataset from stage 1 included complete cardiovascular data from 282 participants, complete cardiac data from 261 participants and complete self-reports from 279 participants. From stage 3, it included complete cardiovascular data from 273 participants, complete cardiac data from 263 participants and complete self-reports from 277 participants. The number of missing data for each variable used in the analysis is presented in the electronic supplementary material, table S6. We used the Mplus default estimation option (i.e. the full-information maximum likelihood) to handle these missing data.

##### 2.5.1.3. Affective data reduction

As the primary measure of positive and negative affective experience, we created latent factors. Since negative and positive emotions are separable [131,132], we created two factors. To account for the nested structure in our data (i.e. measures nested by the participant), we fitted multilevel structural equation models with the condition as the predictor and positive affect and negative affective experiences as the outcomes.

##### 2.5.1.4. Physiological data reduction

We calculated the 60 s ensemble averages from the last minute of the resting baseline and pre-match baselines for physiological measures. We used reactivity scores corrected for the resting-state levels to operationalize physiological changes. Thus, we subtracted the last minute of resting baseline levels from pre-match baselines. Using difference scores is a standard strategy for studying autonomic responses to psychological factors [19,133,134].

Following the approach from the initial study on SMI [9], we used TPR as the primary measure of challenge/threat cardiovascular response. Furthermore, for the robustness check in the secondary analysis, we used the challenge-threat index (CTI) and CO. CTI integrates the TPR and CO information. The CTI is based on the assumption that the TPR and CO are two related measures of the same underlying nervous system activation [3]. Thus, we converted TPR and CO reactivity values into *z*-scores and summed them with an assigned weight of −1 for TPR and 1 for CO. This approach has been used in studies examining the effect of challenge and threat responses on performance outcomes [16,20,35,36]. Greater CTI and CO change scores indicate a greater cardiovascular challenge response.

### 2.5.2. Manipulation checks

Although we ran a series of manipulation checks, we used intention-to-treat analyses. Thus, the data were analysed for all individuals randomized to the condition and provided outcome data, regardless of their adherence to the affect regulation intervention. We used principal component analysis component scores for multi-item scales.

#### 2.5.2.1. Intervention evaluation

To test whether the SMI participants evaluated the intervention and the whole study differently than the control participants, we used *t-*tests with the condition as predictor and acceptability, beliefs and motivation items from laboratory visit 1 as the outcome.

#### 2.5.2.2. Situational affect regulation

To test whether the SMI participants used a reappraisal more often than the control participants, we used *t-*tests with the condition as a predictor and items related to reappraisal from situational affect regulation from the end of stage 3 as the outcomes.

#### 2.5.2.3. Negative appraisals

To test whether the SMI facilitated situational appraisals more strongly than the control condition, we used a *t*‐test with the condition as a predictor and the situational appraisal index as the outcome.

#### 2.5.2.4. Demands and resources evaluations

To test whether the SMI participants appraised gaming performance as a challenge more than the control participants, we included the appraisal ratio in the two-level manipulation check model.

#### 2.5.2.5. Motivated performance

To evaluate whether the participants were engaged in the performance, we used PEP and HR reactivity, which measure sympathetic nervous system activation [3]. A greater PEP decrease and HR increase indicated greater physiological engagement in the performance. To test whether participants were engaged in gaming performance, we ran a two-level manipulation check model with the study phase (resting baseline versus pre-match baseline) as the predictor and PEP and HR as the outcomes.

#### 2.5.2.6. Negative prior mindsets

To test whether the SMI decreased negative mindsets before the tournament more strongly than the control condition, we used *t-*tests with the condition as a predictor and change in fixed and stress mindsets as the outcomes. We calculated the change in fixed and stress mindsets by subtracting the values of stage 1 from stage 3.

### 2.5.3. Primary hypotheses

#### 2.5.3.1. Structural equation model

**Figure 3.**
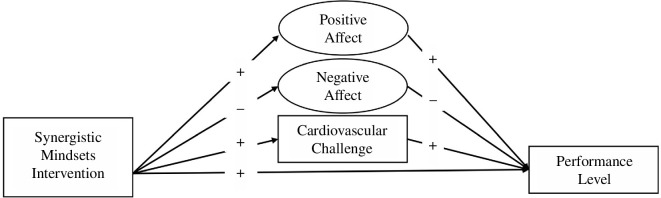
Statistical model for role of affect regulation in esports performance.

In our primary analysis, we focused on two general research questions, namely, does the SMI lead to greater challenge affective response and superior performance compared with control intervention? Specifically, we tested whether the SMI led to more positive and less negative affective experience (RQ1a and b), greater cardiovascular challenge responses (RQ2) and superior performance (RQ3) compared with control condition. We used a two-level structural equation modelling (SEM) approach with an Maximum Likelihood Robust (MLR) estimator, using Mplus 8.0 [77]. We used this method of hypothesis testing in similar gaming projects [19,34]. This technique tests direct and indirect effects between experimental factors (dummy-coded groups with intervention type) and outcomes [135]). We accounted for the non-independence of observations by nesting each round of responses within individuals [136]. The two-level SEM model is presented in figure 3.

We expected that the SMI (versus control) would lead to a greater challenge affective response before the tournament performance in stage 3, operationalized as more positive affective experience (hypothesis 1a), less negative affective experience (hypothesis 1b) and greater challenge cardiovascular response (hypothesis 2). We also expected that the SMI (versus control) would lead to better performance levels during the tournament performance in stage 3 (hypothesis 3).

We tested hypotheses 1a, 1b, 2 and 3 by comparing the intervention types—the synergistic mindsets group versus the control group (table 1). In the models, the intervention type was introduced as a predictor (dummy-coded). Support for the hypotheses would be evident if the models fit the data well, i.e. RMSEA < 0.06; Standardized Root Mean Square Residual (SRMR) < 0.08; Comparative Fit Index (CFI) > 0.95, the *p*-value for the *χ* ^2^ > 0.05 [137]; and the 95% CIs of effect sizes for the regression coefficients for hypothesized paths did not include zero. We evaluated multiple fit indices because evaluating any single index can be problematic (e.g. a significant *χ* ^2^ test does not have to imply the model misfit, as the significance of the test can be affected by many factors, including clustered data, non-normal data big samples; [138–140]). We planned not to interpret effect sizes if the *χ* ^2^ test for model fit and all fit indices suggest model misfit. If the *χ* ^2^ test detects beyond-chance discrepancies between the model and the data (significant *p*‐value), we planned to examine the possible local sources of a causal misfit by examining the matrix of residuals for correlations and modification indices. If the modification indices suggest some small model modifications that also have a theoretical foundation, we planned to include them in the model. Then, if the *χ* ^2^ test still suggests model misfit but: (i) there are no large modification indices and/or residuals, (ii) all other fit indices suggest model fit, and (iii) there are no Haywood cases (e.g. negative variances, standardized coefficients above 1.00), we would conclude that the theoretical model is likely to be close to the observed reality and we would interpret the effect sizes.

We also expected that challenge affective responses would mediate the effects of the SMI (versus control) on performance levels. We planned to test the mediational effects of challenge affective response in three ways (positive and negative affective experience and cardiovascular response). More specifically, we expected that the effects of the SMI (versus control) on better performance levels would be mediated by more positive affective experience (hypothesis 4a), by less negative affective experience (hypothesis 4b) and greater challenge cardiovascular response (hypothesis 5) before tournament performance in stage 3. We planned to test mediational effects because including mediators often increases power relative to testing total effects only [62,64]. Thus, testing mediations decreases the odds of type II error when less pronounced effects are studied.

#### 2.5.3.2. Equivalence tests and minimal effects tests

In the case that the CIs for regression coefficients for hypotheses 1a, 1b and 3 included zero, we planned to use the equivalence test to determine whether the SMI and control intervention had the same effects on participants. Suppose the observed effect lies inside the boundaries of the smallest effect of interest, and the 90% CI around the observed effect does not overlap with the smallest effect of interest. In that case, we would conclude that the synergistic mindsets and control interventions have the same practical effects on gamers. We used this approach because we were unable to recreate the results from the Mplus model in R and, in turn, use the R packages that would allow running equivalence tests. Thus, we used the binary category to infer whether synergistic mindsets and control interventions had the same practical effects.

For hypotheses 1a, 1b and 3, we planned to consider the practical value of the SMI with a minimum effect test. For example, suppose the CI around the observed effect does not overlap with the smallest effect of interest. In that case, we would conclude that synergistic mindsets are a practically beneficial (harmful) approach for gamers. As with the smallest effect size of interest, we aimed to identify changes that are likely to be observed by people and to make an impact in real circumstances; we did not plan to use the same approach for hypothesis 2 because we did not find a way to operationalize the smallest effect of interest for the practical value of cardiovascular responses.

### 2.5.4. Exploratory analyses

#### 2.5.4.1. Moderation analysis

We explored whether other factors could moderate the effect of the intervention by adding to the primary model the moderation of the negative prior mindsets, self-esteem, interoception ability and gaming experience. We decided not to test moderation within the primary model, as we did not find strong enough evidence on whether these factors moderate the effects of the SMI on cardiovascular and performance outcomes [9]. Furthermore, we tested the effects of the SMI when controlling for the differences in intervention evaluations.

#### 2.5.4.2. Quasi-multiverse analysis

We also explored the robustness of our findings by testing alternative operationalizations of the variables used in the model. For positive/negative affect, we used the overall negative and positive affective experiences scores by (i) using principal component analysis component scores for positive and negative experiences, and (ii) averaging raw scores of the four negative or positive affective experiences (as originally intended by the scale [110]), used in a recent large-scale study [69]. For cardiovascular challenge responses, we used CTI or CO from the pre-match baseline or CO from the match instead of TPR. We also used the cardiovascular reactivity scores corrected for the resting-state levels from stage 1 instead of the resting-state levels from stage 3. For performance level, we used the number of kills, deaths and kill-to-death ratio instead of match scores. This analysis aimed to describe the range of effect estimates based on all reasonable data analytical decisions. Finally, in the exploratory analysis, we tested other reasonable SEM models (e.g. one mediational model for affect and a second for the cardiovascular challenge).

Furthermore, our study provided data that might serve future studies for hypothesis formulation. For instance, our data present how appraisals change during positive and negative situations after the SMI and control intervention. The data might also be used to explore methodological (e.g. is there a difference in conclusions when using cardiovascular signals from pre-match baseline compared with match period?), theoretical (e.g. does affect regulation facilitate the cardiovascular recovery from performance?) and practical (e.g. are the effects of the intervention stronger after two weeks than immediately after learning the information?) questions. The electronic supplementary material provides raw data, means and standard deviations.

## 3. Results

### 3.1. Manipulation checks

In this section, we present the results of the preregistered manipulation checks. In the electronic supplementary material, table S4, we present the results for all scales included throughout the project, namely before laboratory visit 1, before laboratory visit 2, between laboratory visits and one month after laboratory visit 2. Here, we refer to participants who received SMI and control intervention as the SMI and control participants.

#### 3.1.1. Intervention evaluation

The SMI participants (T1: *M* = 55.88, s.d. = 4.54; T2: *M* = 54.08, s.d. = 6.25) compared with the control participants (T1: *M* = 52.81, s.d. = 6.54; T2: *M* = 49.76, s.d. = 8.21) evaluated the intervention they received more positively after the first laboratory visit (T1) *t*_254.45_ = −4.88, *p* < 0.001, *d* = 0.58, 95% CI [0.34, 0.81] and after the second laboratory visit (T2) *t*_185.96_ = −4.40, *p* < 0.001, *d* = 0.62, 95% CI [0.34, 0.90].

#### 3.1.2. Situational affect regulation

The SMI participants (*M* = 4.50, s.d. = 1.87) compared with the control participants (*M* = 3.60, s.d. = 2.02) reported more frequent usage of reappraisal during negative situations in the tournament *t*_286.81_ = −3.95, *p* < 0.001, *d* = 0.47, 95% CI [0.23, 0.70]. However, the SMI participants (*M* = 3.18, s.d. = 1.84) compared with the control participants (*M* = 2.88, s.d. = 1.95) did not report more frequent usage of reappraisal during positive situations in the tournament *t*_288.18_ = −1.36, *p* = 0.17, *d* = 0.16, 95% CI [−0.07, .039].

#### 3.1.3. Negative appraisals

The SMI participants (*M* = 9.86, s.d. = 3.11) compared with the control participants (*M* = 10.88, s.d. = 4.06) appraised the tournament in a significantly less negative way *t*_271.92_ = 2.21, *p* = 0.03, *d* = −0.26, 95% CI [−0.49, −0.03].

#### 3.1.4. Demands and resources evaluations

The SMI participants (*M* = 1.57, s.d. = 2.38) appraised the upcoming gaming performance similarly to the control participants (*M* = 1.40, s.d. = 2.26) *β* = 0.17, *t*_288.99_ = 0.68, *p* = 0.50.

#### 3.1.5. Motivated performance

The players' PEP baseline levels (*M* = 117.10 ms, s.d. = 11.73) compared with the pre-match levels (*M* = 117.67 ms, s.d. = 11.88) was lower significantly *β* = 0.55, *t*_2232.50_ = 0.68, *p* = 0.04. Players' HR baseline levels (*M* = 79.25 bpm, s.d. = 11.41) compared with the pre-match levels (*M* = 78.43 bpm, s.d. = 11.14) did not differ significantly *β* = −0.77, *t*_2307.95_ = 3.50, *p* < 0.001.

These results might indicate that players were not engaged in the performance. However, in the exploratory analysis, we examined the range of PEP across the matches and observed stable increases (decreases) within the PEP (HR) levels. This might suggest that comparing the resting baseline and all pre-match baselines might not be the optimal statistical approach owing to the elevated baseline level. Thus, as part of the exploratory analysis, we compared the resting baseline levels with the first pre-match levels. Players' first pre-match levels (*M* = 116.03 ms, s.d. = 12.03) compared with the PEP baseline levels (*M* = 117.10 ms, s.d. = 11.73) was significantly lower *β* = −1.10, *t*_281.15_ = −3.64, *p* < 0.001. Similarly, pre-match levels (*M* = 80.32 bpm, s.d. = 11.55) compared with the players' HR baseline levels (*M* = 79.25 bpm, s.d. = 11.41) was significantly higher β = 1.07, *t*_289.00_ = 4.42, *p* < 0.001. Thus, we decided to interpret the physiological indicators of challenge and threat.

#### 3.5.6. Negative prior mindsets

The SMI participants (*M* = −2.67, s.d. = 4.11) compared with the control participants (*M* = −1.18, s.d. = 3.27) displayed significantly greater decreases in stress mindsets *t*_270.22_ = 3.35, *p* < 0.001, *d* = −0.40, 95% CI [−0.63, −0.16]. Furthermore, the SMI participants (*M* = −0.67, s.d. = 3.65) compared with the control participants (*M* = 0.07, s.d. = 3.52) displayed greater, but not significant decreases in fixed mindsets *t*_287.41_ = 1.81, *p* = 0.07, *d* = −0.21, 95% CI [−0.44, 0.02].

### 3.2. Primary analysis

#### 3.2.1. Structural equation model

**Figure 4.**
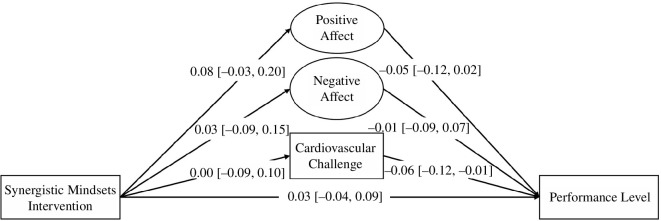
Structural model for role of affect regulation in esports performance.

The path model for the role of affect regulation in esports performance is presented in figure 4. Descriptive statistics and correlations are presented in the electronic supplementary material, table S5. This model presented mediocre fit to the data, $\chi_{37}^{2}$ = 142.12, *p* < 0.001, RMSEA = 0.04, 90% CI [0.03, 0.04], CFI = 0.94.

The SMI (versus control) did not lead to a greater challenge affective response before the tournament performance in stage 3, operationalized as a lack of more positive affect *β* = 0.08, 95% CI [−0.03, 0.20] (hypothesis 1a), a lack of less negative affect *β* = 0.03, 95% CI [−0.09, 0.15] (hypothesis 1b), a lack of greater challenge cardiovascular response (TPR reactivity) *β* = 0.00, 95% CI [−0.09, 0.10] (hypothesis 2). Furthermore, the SMI (versus control) did not lead to better performance levels during the tournament performance in stage 3 *β* = 0.03, 95% CI [−0.04, 0.09] (hypothesis 3).

The way participants felt before matches did not influence the performance results (for positive affect, *β* = −0.05, 95% CI [−0.12, 0.02]; for negative affect *β* = −0.01, 95% CI [−0.09, 0.07]. Participants who displayed decreased cardiovascular challenge (stronger TPR increases) performed better *β* = −0.06, 95% CI [−0.12, −0.01]. We did not find any significant mediational effects of the SMI on performance compared with the control condition (hypotheses 4a, 4b, 5). Detailed output from Mplus is presented in the electronic supplementary material, Mplus outputs model 1.

#### 3.2.2. Equivalence tests and minimal effects tests

As we determined the effects of interest to lie outside the interval from −0.22 to 0.22, we computed 90% CIs and compared these to the equivalence range. If the observed CI falls completely within the equivalence range, we can statistically reject the presence of effects large enough to be of interest. If the 90% CI would fall completely outside the equivalence interval, we cannot just reject the null hypothesis, but also conclude the observed effect is statistically large enough to be of interest (i.e. a minimal effect test). We observed that the SMI and control intervention had the same practical effects on participants in terms of how much positive affect they felt *β* = 0.08, 90% CI [−0.01, 0.17] versus SESOI (−0.22, 0.22), how much negative affect they felt *β* = 0.03, 90% CI [−0.07, 0.14] versus SESOI (−0.22, 0.22), and how well they performed *β* = 0.03, 95% CI [−0.03, 0.10] versus SESOI = (−0.22, 0.22). As the observed confidence intervals fall completely within the equivalence range, we statistically rejected the presence of effects large enough to be of interest.

### 3.3. Exploratory analyses

#### 3.3.1. Moderation analysis

We did not observe the moderation of the negative prior mindsets, self-esteem, interoception ability and gaming experience on the effects of the SMI on gaming performance, positive and negative affect (see the electronic supplementary material, Mplus outputs models 73–77, for details). Furthermore, testing the effects of the SMI when controlling for the differences in intervention evaluations did not change the conclusions from the model (see the electronic supplementary material, Mplus outputs models 78, for details).

#### 3.3.2. Quasi-multiverse analysis

Using different operationalizations of variables did not influence the conclusions from the models. With 72 tested models (see the electronic supplementary material, QuasiMultiVerse.xlsx, and Mplus outputs models 1–72, for details), we found that the effects ranged from 0.07 to 0.08 for the effect of the condition on positive affect, from 0.03 to 0.04 for the effect of the condition on negative affect, from −0.07 to 0.00 for the effect of the condition on challenge-type cardiovascular response, and from 0.02 to 0.04 for the effect of the condition on participants performance. Furthermore, the strength of associations between positive affect and score ranged from −0.05 to −0.02, between negative affect and score ranged from −0.02 to 0.00, and between challenge-type cardiovascular response and score ranged from −0.06 to 0.05. All the presented effects did not overlap with the SESOIs, which implies we can reject the presence of effects we deemed large enough to be of interest for this study.

## 4. Discussion

This project examined the potential of modifying affective responses to optimize esport performance. We adapted SMI to the high-stakes esports context and examined whether and how it influences performance outcomes. The SMI was well received by participants and led to the adoption of more beneficial stress mindsets, more positive appraisals of the esports tournament and increased use of reappraisal emotion regulation strategies. However, we observed no effects of the intervention on affective responses or performance in gaming contexts. One possible explanation for these effects is that we found that the high-stakes esports competition was a *positive* experience rather than a potentially negative evaluative stressor, which limited the opportunity for the SMI to exert a meaningful effect on affective and physiological responses. That is, there was no negative physiological stress response for the intervention to regulate. Together, these findings point to important questions about the fit of such an intervention in diverse performance settings with varying levels of stress.

### 4.1. Sculpting appraisals using synergistic mindsets intervention

This study aimed to advance affective science in several ways. First, we integrated emotion experiences and stress responses as affective responses [33]. Although stress responses and emotions are often viewed as separate phenomena, they both involve appraisals and whole-body reactions to psychologically relevant situations [3,30–33]. Our integration allowed us to capture gamers’ affective states—the way people evaluate and feel in esports competition—more precisely.

Second, we advanced previous performance-related studies that used relatively brief and focal (i.e. single appraisal-oriented) interventions (e.g. [16,17,21,34–37,42,43]); with a 30–40 min single-session SMI. We replicated some effects of the initial sequence of studies [9] and found that the SMI changed mindsets and appraisals. In addition, our adaptation, which included additional parts related to affect regulation, led gamers to use reappraisal regulation strategies more often and over time. Thus, the SMI may be beneficial in the modification of general-level, time-stable beliefs. The observed ability to change mindsets and appraisals is in line with the synergistic, growth and stress-can-be-enhancing mindsets models [4,5,9,56,57] as well as the broader arousal reappraisal model [2]. The observed effects may have implications for mental health and well-being. The cultivation of beneficial stress mindsets and more positive tournament appraisals reflects a potential pathway for enhancing athletes' mental resilience and adaptive coping strategies in competitive settings [141,142]. Furthermore, the increased use of reappraisal emotion regulation techniques suggests that the SMI can provide individuals with valuable tools for effective emotion management. Because we found no evidence that the SMI causes harm and some evidence that it can have important benefits, our study supports the claims of the original study that the SMI is the prevention tool that is recommended because of its ease of implementation and scalability. Even small changes resulting from the SMI that harness these psychological adaptations have implications for broader applications to promote long-term mental health.

The design of this study led us to believe that participants would change not only their mindsets but also their esports-specific situational appraisals and affective evaluations. Although participants went through the SMI version adapted to the gaming context and were asked to apply the knowledge and techniques learned to their daily gameplay, the SMI did not influence preregistered and hypothesized challenge/threat affective responses. Thus, our performance-specific test of the practical use of the SMI failed to have demonstrable downstream affective and performance consequences in this context.

One explanation for the observed pattern of data is that the esports competition context used in this study was not stressful, limiting the potential of the SMI to exert effects on performance or *in vivo* stress responses. Supporting this interpretation, participants did not display threat-type physiological responses before the matches, as is typically observed in other evaluative- and competition-based paradigms studying the effects of stress optimization manipulations (e.g. [9,45,51,54]). That is, participants reported less experienced negative stress than any of the positive emotions before matches, indicating that esports competition with cash prizes was a positive and exciting event and, hence, not an ideal target for intervention. This indicates that our assumption that organizing an esports competition in a laboratory with cash prizes is enough to induce threat-type stress responses in players was wrong and misguided in its current iteration. This might present the situation where the SMI participants learned and practised how to optimize the stress responses but were not able to implement it during the study.

In light of our findings, we explored existing literature on affective responses preceding sports and esports performances. Although comparisons across studies are challenging owing to differing scales, research indicates that esports players generally report low-to-moderate stress levels prior to gaming sessions (37% of the maximum value of given scale [143]; 40% [144]; 38% [145]). This is in line with findings from other sports, such as swimming (20%) [146] and judo (55%) [147]. Slightly higher levels were found for esports players when they were asked to recall recent stress-inducing events (64%) [148]. Anxiety research within the esports domain suggests that players experience medium levels of pre-performance anxiety (from 40% to 66%) [149–151]. Similarly, other athletes report low to medium levels of anxiety before the performance (from 30% to 55%) [16,17,35,152]. However, the interpretation of felt anxiety circles around neutrality with very slight pleasant and unpleasant interpretations [16,17,35]. These findings align with studies in which athletes experienced more positive emotions (e.g. excitement and happiness) before the performance than negative emotions (e.g. anxiety [71,72,153]). Finally, we found that the levels of heart rate for other esports studies ranged from around 70–80 bpm [19,34,145,154,155] to around 90–100 bmp before gaming [156,157].

These findings suggest that the pre-performance situation is characterized by low to moderate levels of stress that coexist with positive affective states. Thus, the context of our study aligns with normal affective responses observed in laboratory settings, indicating our study’s context did not deviate from established norms. This raises an important question for researchers: should sports performance be considered a conventional stress scenario where existing affective models are applicable, or is it a distinct context that necessitates adjusting, validating or even developing new, context-specific affective models? This debate underscores the need for a nuanced understanding of affect in sports, encouraging a re-evaluation of how we conceptualize and address the interplay of stress and positive emotions in performance settings.

### 4.2. Optimizing performance using synergistic mindsets intervention

The findings observed here were in contrast to previous studies that have shown that both situation reappraisals and response reappraisals can help optimize performance outcomes, including motor [16,17,35,36,49] and cognitive tasks [38–40,43,50–52], artistic performance [43] and public speaking [42,43]. Although our project provided a greater opportunity to demonstrate the positive effects of reappraisal-based interventions (e.g. we not only introduced the concept but also allowed participants to train using reappraisal), findings did not support the expectation that this intervention would impact game performance.

One explanation for such effects is the pivotal role of context in shaping affective responses [158]. Thus, the findings do not necessarily have to be in contrast to previous studies and theoretical models (i.e. synergistic mindsets model [9], the growth mindset model [56,57]; the arousal reappraisal model [2]; the stress-can-be-enhancing mindset model [4,5]) but might present the relationship between the performance outcomes and appraisals within the context of the overall positively evaluated competition event. Previous work shows how diverse contexts can fundamentally shape affective responses (see [158] for the review), suggesting that the differential nature of performance contexts may have interacted with the intervention effects. Our study aligns with this understanding, emphasizing the relevance of context in affective responses and performance outcomes and highlighting the need for nuanced context-specific investigations within affective science.

Another explanation for such effects is that sports performance requires the execution of well-trained skills and knowledge, and it may be naive to expect that people’s performance will change after a brief intervention. However, this perspective would be very pessimistic for most experimental studies in sports and performance psychology, as most of the previous studies have used briefer interventions and yet have found impacts on performance outcomes [16,17,21,34–37,42,43].

### 4.3. Role of affective responses in optimizing performance

We designed our study not only to test the effects of the SMI on performance but also to examine the mechanism by which it might have its effects. We chose esports, in which gamers compete in a seated position in front of the screen, which provides an excellent opportunity to examine affective responses related to performance in a laboratory setting [19,34]. However, contrary to expectations, prior research (see [3]for a review) and the biopsychosocial model of challenge and threat [3], threat-type cardiovascular responses were loosely related to *better* performance, but the quasi-multiverse analysis indicated that this conclusion depended on how challenge/threat cardiovascular responses were operationalized. Overall, the data presented here did not support the adaptiveness (maladaptiveness) of the challenge (threat) cardiovascular response [3,20,26].

Importantly, performance efficiency did not depend on whether they felt positive or negative before the matches. Thus, we did not replicate basic, well-established links between positive affect and better performance [19,23,25,27,29]; or between negative affect and worse performance [22–25]. The observed effects were smaller than the literature would suggest. Our findings, however, are consistent with other work highlighting the ambiguity of the relationship between affect and performance ([71,72,159,160]; see [8] for the review). In exploratory moderation analyses, we found no evidence that the same emotion could be functional or dysfunctional depending on the individual’s evaluation and beliefs [161].

Our study also introduced a quasi-multiverse analysis to investigate whether the conclusions from the study depend on the variables’ operationalizations. When we used different operationalizations (e.g. latent factor, average or Principal Component Analysis score or the average of four items for positive affect), we found little variation in the associations between affective experiences, cardiovascular responses and esports performance outcomes. This is an optimistic message for affective science, as in some situations, researchers may not be able to use optimal measures (e.g. measuring TPR during gaming). We hope to see this kind of analysis more often in the affective literature, as it helps to assess the robustness of findings.

### 4.4. Limitations and future directions

The study has several limitations that bear noting when interpreting results.

First, we focused on a single performance context—esports. Esports competitions present similar psychological demands compared with traditional gross motor sports, but, unlike traditional sports, are characterized by remaining seated and less physical exertion. This may limit the generalization of our results, especially those tied to physiological responses, to other sports contexts. Future research could explore the impact of the SMI in other sports contexts to better understand the links between specific affect and performance-related outcomes.

Second, the esports tournament context in our study did not elicit high levels of negative evaluative stress. Rather, the prevalent affective state before the matches leaned more towards positive than negative affect. This contextual aspect, combined with the absence of affect manipulation by SMI, limits the comprehensive assessment of the performance-optimizing potential of SMI. Future research might aim for even greater ecological validity that may provide a more stressful study context. Researchers might add to the laboratory experiments other pressure-eliciting elements of sports tournaments, including punishment contingency, openly published leader board, comparison with professional players, live streaming, in-person audience and feedback in between matches [151,162–165]. Ideally, the researchers should aim to collect data during actual tournaments.

Third, our sample was quite homogeneous: participants were all young males and primarily recreational yet experienced gamers. In games like first-person shooters, males make up most of the player base [166]. Our latest study found that only 7% of CS: GO players in Poland were women [167]. As this gender balance changes, we hope future studies will focus on the relationship between affect and performance among professionals and female gamers. Moreover, young males who self-select themselves into gaming as a recreational activity may not be an ideal target group for intervention in gaming contexts. Stress optimization interventions like the SMI are most effective for people who do not already hold positive, challenge-type stress appraisals, otherwise, the intervention is ‘teaching people what they already know’. For example, in the first sequence of the SMI studies, those who benefited the most from the SMI held negative prior mindsets about stress and growth [9]. Thus, if non-experienced gamers were recruited in follow-up research, the heightened uncertainty and (presumably) lower resource appraisals would be more likely to lead to threat-type responses that could be regulated.

Fourth, for this project, we adapted two validated interventions to the esports performance context [9,94]. As our work aimed to answer the initial study authors’ call for new large-scale trials in diverse populations and contexts [9], we made only essential changes and added cover stories to minimize differences between conditions. Despite our efforts, we observed differences between the conditions in how they were evaluated. Future studies might aim to create new control interventions that will resemble the active conditions more closely or include topics closely related to a given activity (e.g. materials on the history of esports, game strategy and biomechanics of movements).

Fifth, to determine the robustness of our results and their practical effects, we used quasi-multiverse analysis. Existing software solutions did not allow us to use the regular test. Thus, we formulated conclusions based only on these approaches’ guiding principles, making dichotomous decisions. As the progress in statistical and software solutions is an inseparable part of scientific progress, researchers in the future might use our open data to re-examine our findings.

The last limitation of our study relates to the fit of our proposed model to the empirical data. Despite the modest sample size and the low magnitudes of inter-relationships among variables considered, the *χ* ^2^ test indicated a less-than-optimal fit, with the value being nearly four times as large as expected. This discrepancy suggests potential model-data deviations that might not be entirely attributed to random sampling variability. Furthermore, our model’s Comparative Fit Index (0.94) did not meet the lenient benchmark of 0.95 advocated by [168], suggesting that the approximate fit of our model could be considered mediocre. These findings imply that while our model offers some insights, it may not fully capture the complexities of the underlying phenomena studied. Our study’s findings should, therefore, be interpreted with caution, recognizing that the model’s fit to the data is an area for improvement and further investigation.

## 5. Conclusions

This study tested the practical use of the SMI for esports. Notable strengths of this study include using a multi-stage experimental approach with a large sample of highly motivated individuals in an esports performance context. Furthermore, following the open science recommendations and preregistration, we tested the effects rigorously. We found that the SMI was well received by participants and led to the adoption of more beneficial stress mindsets, more positive appraisals of the esports tournament and increased use of reappraisal emotion regulation strategies. However, we found the minimal impact of the SMI on the performance outcomes and challenge/threat affective responses. Furthermore, our findings question the previous assumptions about the relationship between affective responses and performance outcomes. In summary, the findings lead us to recommend the SMI as a prevention tool but not as a performance-enhancing intervention in the context of a positively evaluated performance.

## Data Availability

All data used for analysis and materials are available on the Open Science Framework (OSF) website: [169]. We will also share all raw data, videos from the lab sessions, and daily reports as the openly available dataset. Code Availability: All analysis code (completed in Mplus and R) is available on the Open Science Framework (OSF) website [169]. Electronic supplementary material is available online at [170].

## Appendix A

Stage 1 recommendation and review history: https://rr.peercommunityin.org/articles/rec?id=364.

Stage 2 recommendation and review history: https://rr.peercommunityin.org/articles/rec?id=712.
